# Young Infants Clinical Signs Study 8-sign Algorithm for Identification of Sick Infants Adapted for Routine Home Visits: A Systematic Review and Critical Appraisal of its Measurement Properties

**DOI:** 10.1177/2333794X231219598

**Published:** 2024-01-25

**Authors:** Alastair Fung, Julie Farmer, Cornelia M. Borkhoff

**Affiliations:** 1 Hospital for Sick Children, Toronto, ON, Canada; 2 University of Toronto, Toronto, ON, Canada; 3 Hospital for Sick Children Research Institute, Toronto, ON, Canada

**Keywords:** clinical algorithm, community health worker, measurement properties, developing country, young infants

## Abstract

*Objective.* The 8-sign algorithm adapted from the Young Infants Clinical Signs Study (YICSS) is widely used to identify sick infants during home visits (YICSS-home algorithm). We aimed to critically appraise the development and evidence of measurement properties, including sensibility, reliability, and validity, of the YICSS-home algorithm. *Methods.* Relevant studies were identified through a systematic literature search. *Results.* The YICSS-home algorithm has good sensibility. The algorithm demonstrated at least moderate inter-rater reliability and sensitivity ranging from 69% to 80%. However, the algorithm was developed among sick infants brought for care to a health facility and not initially developed for use by community health workers (CHWs) during home visits. Some important risk factors were omitted at item generation. Inter-CHW reliability and construct validity have not been estimated. *Conclusion.* Future research should build on the strengths of the YICSS-home algorithm and address its limitations to develop a new algorithm with improved predictive accuracy.

## Introduction

In 2020, 2.4 million children worldwide died in the neonatal period (0-28 days of age).^1^ An estimated 98% of neonatal deaths occur in low- and middle-income countries (LMICs).^2^ Moreover, a substantial proportion of these deaths occur at home.^3^ As such, in resource-limited settings with poor access to hospital-based care, home-based interventions to reduce infant mortality have been implemented including community health worker (CHW) postnatal home visits.^4^ Identification of potentially life-threatening illnesses among young infants (0-59 days of age) by CHWs during home visits and subsequent referral to hospital are critical to reducing infant mortality in LMICs.

**Figure 1.**
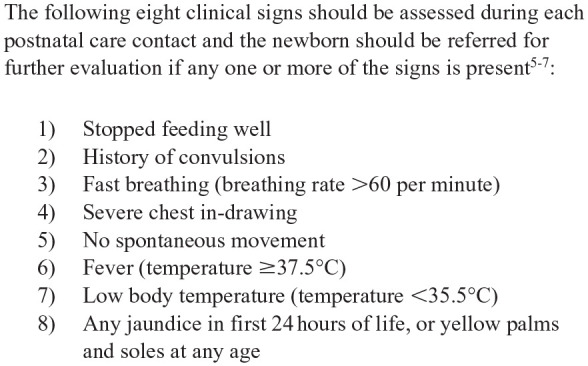
YICSS-home algorithm.

World Health Organization (WHO) postnatal care guidelines^5-7^ recommend an 8-sign algorithm for illness recognition when assessing young infants during routine home visits. This algorithm consists of the 7-sign Young Infants Clinical Signs Study (YICSS)^8^ algorithm applied to the home visit setting with the addition of jaundice as the eighth sign. It is hereafter referred to as the YICSS-home algorithm (Figure 1). Since laboratory and imaging investigations are rarely available in LMIC community settings,^9,10^ the algorithm relies exclusively on history and physical examination. Eight clinical signs should be assessed during each home visit and an infant should be referred for further evaluation if any one or more of the signs is present.

The clinical signs from the YICSS-home algorithm have been widely implemented in LMIC research studies evaluating the impact of CHW home visit programs on newborn survival.^4,11-14^ The signs are also used to define a clinical diagnosis of possible serious bacterial infection (pSBI) in studies estimating the incidence of pSBI in infants in LMICs.^15,16^

In 2014, WHO newborn health research priorities included investigating whether simple clinical algorithms can be used by CHWs to identify and refer neonates with signs of infection and thereby reduce newborn mortality.^17^ Uptake of this priority has been moderate to date.^18^ To address such priorities and before adopting a measurement tool in any study, it is important to critically appraise the quality of its development and evidence of its measurement properties for its intended use. No previous publication has reviewed the measurement properties of the YICSS-home algorithm, and thus a focused critical appraisal is lacking in the literature. We aimed to critically appraise the development, sensibility, reliability and validity of the YICSS-home algorithm to measure risk of severe illness or death among young infants assessed by CHWs during home visits.

## Methods

This manuscript followed the Preferred Reporting Items for Systematic Reviews and Meta-Analyses (PRISMA) checklist (Supplemental Table 1).^19^ No protocol was prepared or registered prior to conducting the review.

### Search Strategy and Information Sources

We searched MEDLINE, Embase and CINAHL for all relevant articles from their inception to July 2022. Search terms reflected the concepts of infants, clinical algorithms, CHWs, severe illness, measurement properties and LMICs (Supplemental Table 2). We applied the COSMIN search filters for measurement properties for all 3 databases.^20^ We also hand searched the reference lists of the YICSS,^8^ which is the sentinel publication describing the development of the YICSS-home algorithm, and the reference lists of relevant reviews on clinical signs to identify severe infant illness in LMICs.

### Eligibility Criteria

Eligible studies included any primary study in English that reported on the development or measurement property testing of the YICSS-home algorithm to predict a severe illness or death in young infants. Inclusion criteria were: (1) infants 0-59 days of age; (2) conducted in the home visit setting in a LMIC; (3) the algorithm was applied by a CHW; (4) the outcome or criterion was a severe illness or death; and (5) reported on the development, reliability, criterion validity and/or construct validity of the 8-sign YICSS-home algorithm or variations of the algorithm that included at least 5 of the 8 signs (Figure 1). We used the World Bank definitions for determining LMIC status.^21^ We defined CHWs as individuals who: (i) have some training in functions related to delivering biomedical health care; (ii) have no formal professional certificate; and (iii) are paid or volunteer.^22,23^ We defined severe illness as requiring referral and/or admission to hospital or a serious bacterial infection including urinary tract infection, pneumonia, sepsis, bacteremia or meningitis. We excluded conference abstracts, dissertations/theses, review articles, study protocols, and commentaries.

### Study Selection and Data Extraction

Two authors (AF and JF) independently performed the eligibility assessment for each article using the inclusion and exclusion criteria first in abstract form followed by full-text format. Differences were resolved through discussion between the 2 authors. Cohen’s kappa statistic was calculated between the 2 authors.

One author (AF) extracted the following information from each included study: Author name, year, country, age group, study setting, study design and objectives, type of assessor, criterion or gold standard (if applicable), instrument development information, methods and evidence of reliability and validity. A second author (JF) performed an audit of data extraction and synthesis tables for accuracy and completeness.

### Measurement Properties and Synthesis

We evaluated the development of the YICSS-home algorithm by appraising item generation and item reduction. We used Feinstein’s^24^ framework to evaluate sensibility, which includes (1) Purpose and framework, (2) Comprehensibility, (3) Replicability, (4) Suitability of scale, (5) Face validity, (6) Content validity, and (7) Ease of usage. Lastly, we summarized the evidence of the YICSS-home algorithm’s reliability, criterion validity and construct validity.

### Ethical Approval and Informed Consent

Ethical approval and/or informed consent was not required for this review article. No data was collected from human subjects and we used published studies.

## Results

**Figure 2.**
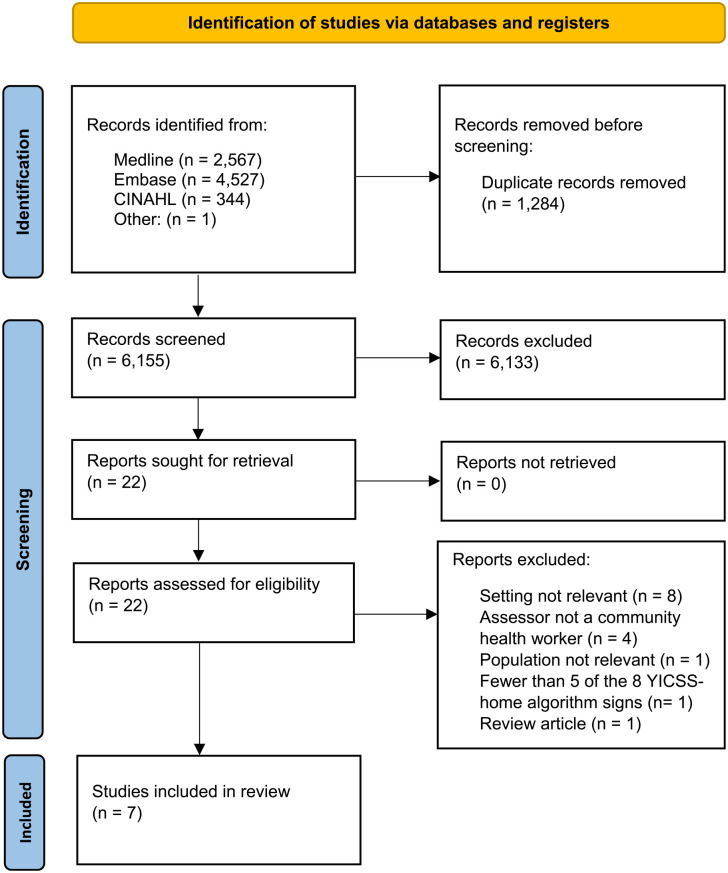
Summary of literature search and review for eligible studies.

Our systematic literature search results are summarized in Figure 2. Of the 6155 citations identified after duplicates were removed, 22 were identified as potentially eligible and were retrieved for full-text review. After review, 15 studies were excluded. Reasons for exclusion are provided in Supplemental Table 3. Cohen’s kappa statistic between the 2 authors at the title and abstract stage was moderate (0.49, 95% confidence interval (CI) 0.30, 0.68).^25^ At the full-text stage, kappa was almost perfect (0.91, 95% CI 0.74, 1.00). Seven studies met full inclusion criteria.^8,14,26-30^

Key characteristics of the 7 included studies are shown in Table 1. All studies were published between 2008 and 2014, and sample sizes ranged from 208 to 8889 participants. Most were observational studies and 2 studies involved a secondary analysis of a randomized controlled trial. One study informed the development of the YICSS-home algorithm.^8^ Five studies provided evidence of the algorithm’s reliability.^14,26-28,30^ Three studies assessed the algorithm’s validity.^14,28,29^ Evidence of inter-rater reliability and validity are summarized in Tables 2 and 3, respectively.

**Table 1.** Characteristics of Studies That Reported on the Development, Reliability and/or Validity of the YICSS-Home Algorithm.

Study	Country	Sample size for relevant measurement component(s) (N)	Age group (days)	Setting	Study Design	Measurement components
Young Infants Clinical Signs Study Group^8^	Bangladesh, Bolivia, Ghana, India, Pakistan and South Africa	3177 (aged 0-6 days); 5712 (aged 7-59 days)	0-59 (0-6 and 7-59)	Brought for care to health facilities	Observational study	**Development**: Measured the sensitivity, specificity and odds ratio of a list of 31 symptoms and signs individually and combined into algorithms for prediction of severe illness requiring hospital admission. Derived a 7-sign algorithm used by trained primary health workers that identified infants requiring hospital-level care using pediatrician assessment as the gold standard. The algorithm performed with sensitivity 85% and specificity 75% in infants 0-6 days of age and sensitivity 74% and specificity 79% in infants 7-59 days of age compared to pediatrician assessment.
Baqui et al^26^	Bangladesh	288	0-7	Routine home visits	Observational study	**Reliability**: Measured the level of agreement between CHW and study physician assessments using a 20-sign algorithm that included the YICSS-home algorithm signs.
Darmstadt et al^27^	Bangladesh	395	0-8	Routine home visits	Observational study	**Reliability**: Measured the level of agreement between CHW and study physician assessments using a 35-sign algorithm that included the YICSS-home algorithm signs.
Darmstadt et al^28^	Bangladesh	395	0-8	Routine home visits	Observational study	**Reliability**: Measured the level of agreement between CHW and study physician assessments using the YICSS-home algorithm. **Validity**: Validated several clinical algorithms including the YICSS-home algorithm used by CHWs to identify neonatal illness requiring referral.
Khanal et al^30^	Nepal	653	0-60	Routine home visits	Pilot feasibility study	**Reliability**: Measured the level of agreement between FCHV and FB-CHW for signs of possible severe bacterial infection (that included 6 of the 8 YICSS-home algorithm signs).
Ansah Manu et al^14^	Ghana	759	0-7	Routine home visits	Secondary analysis of a cluster randomized controlled trial	**Reliability**: Measured the level of agreement between CBSVs and DiPS for 6 of the 8 YICSS-home algorithm signs. **Validity**: Measured sensitivity and specificity of CBSV assessments and referrals compared to DiPS assessments and referrals
Gill et al^29^	Zambia	208	0-28	Routine home visits	Secondary analysis of a cluster randomized controlled trial	**Validity**: Measured the sensitivity and specificity of individual signs (that included 6 of the 8 YICSS-home algorithm signs) for prediction of death. Validity of a combination of signs as an algorithm or index was not assessed.

Abbreviations: CBSV, community-based surveillance volunteers; CHW, community health worker; DiPS, district-based project supervisor; FCHV, female community health volunteer; FB-CHW, facility-based community health worker; YICSS, Young Infants Clinical Signs Study.

**Table 2.** Evidence of Inter-Rater Reliability.

Study	Assessor(s)	Item-item agreement/reproducibility	Overall agreement/reproducibility
Ansah Manu et al^14^	CBSV	Kappa statistics of agreement between CBSV and DiPS for each YICSS-home algorithm sign:^a^ • Chest in-drawing (0.85) • Only moves when stimulated (1.00) • Yellow soles (0.84) • Respiratory rate -first count (0.75) • Respiratory rate -second count (0.83) • Hypothermia (0.94) • Fever (0.90)	Kappa statistic of agreement between CBSV and DiPS for referral: 0.87
Baqui et al^26^	CHW	Kappa statistics of agreement between CHW and physician for each YICSS-home algorithm sign:^a^ • “Fast breathing” (0.86) • “Hypothermia” (0.80) • “Fever” (0.80) • “History of convulsion” (0.67) • “Not able to feed or not suck at all” (0.70) and • “Jaundiced palm and sole after 1 day of birth” (0.70)^b^	Kappa statistic of agreement between CHW and physician for “Very severe disease”:^c^ 0.85
Darmstadt et al^27^	CHW	Kappa statistics of agreement between CHW and physician for each YICSS-home algorithm sign:^a^ • “Respiratory rate ≥70” (0.33) • “Respiratory rate ≥60-69” (0.08) • “Severe fever: temperature>38.3°C” (0.67) • “Moderate fever: temperature 37.8-38.3°C” (0.39) • “Severe hypothermia: temperature <35.3°C” (0.66) • “Moderate hypothermia: temperature 35.3-36.4°C” (0.16) • “Unable to feed or suck, or not attached” (0.50) • “Jaundiced palms and soles after the day of birth” (0.32)^d^	Kappa statistic of agreement between CHW and physician for classification of “Very severe disease”:^e^ 0.63
Darmstadt et al^28^	CHW	Not assessed	Kappa statistic of agreement between CHW and physician administering the YICSS-home algorithm: 0.46
Khanal et al^30^	FCHV	Kappa statistics of agreement between FCHV and FB-CHW for each YICSS-home algorithm sign:^a^ • Fever (0.85) • Hypothermia (0.84) • Unable to feed (0.82) • Fast breathing (0.80) • Severe chest in-drawing (0.77) • Lethargic or unconscious (0.74)	Not assessed

Abbreviations: CBSV, community-based surveillance volunteers; CHW, community health worker; DiPS, district-based project supervisor; FCHV, female community health volunteer; FB-CHW, facility-based community health worker; YICSS, Young Infants Clinical Signs Study.

a Inter-rater reliability among CHWs or physicians was not assessed.

b Kappa statistics could not be calculated for “Severe chest in-drawing” and “Unconscious” since these signs had no cases identified by CHWs.

c “Very severe disease” was defined as any 1 of 8 signs, 6 of which are similar or identical to 6 signs found in the YICSS-home algorithm (“Observed convulsions,” “Unconsciousness,” “Fast breathing,” “Severe chest in-drawing,” “Fever,” and “Low body temperature”) and 2 signs not included in the YICSS-home algorithm (“Skin pustules” and “Umbilical redness”).

d Kappa statistics could not be calculated for “Severe chest in-drawing,” “Unconscious” and “Convulsion” since the frequency of these signs was 0%.

e “Very severe disease” was defined as any 1 of 11 signs, 7 of which are similar or identical to 7 signs found in the YICSS-home algorithm (“Observed convulsions,” “Unconscious,” “Fast breathing ≥70 bpm,” “Severe chest in-drawing,” “Fever >38.3°C,” and “Low body temperature <35.3°C”) and 4 signs not included in the YICSS-home algorithm (“Many or severe skin pustules or blisters, or single large area of pus or redness with swelling,” “Umbilical redness extending to the skin,” “Weak, abnormal or absent cry” and “Lethargic or less than normal movement”).

**Table 3.** Evidence of Criterion Validity.

Study	Assessor	Gold Standard	Criterion Validity
Ansah Manu et al^14^	CBSV	DiPS assessment determining that the infant required referral	An 8-sign algorithm, that included 6 of the 8 YICSS-home algorithm signs, used by CBSVs identified infants requiring referral with a sensitivity of 79.5% and specificity of 100% compared to DiPS.
Darmstadt et al^28^	CHW	Physician assessment determining that the infant required referral	The YICSS-home algorithm used by CHWs identified infants requiring referral with a sensitivity of 68.8% and specificity of 95.3% compared to physician assessment.
Gill et al^29^	Traditional birth attendants	Death	Sensitivity and specificity, presented as percentage (95% CI), of 6 of the 8 YICSS-home algorithm signs used by traditional birth attendants: • Fever or felt hot: Sensitivity 15.0 (3.2, 37.9); • Specificity 55.9 (48.3, 63.3) • Refusing to feed: Sensitivity 45.0 (23.1, 68.5); Specificity 91.6 (86.6, 95.2) • Sleepy or difficult to rouse: Sensitivity 20.0 (5.7, 43.7); Specificity 97.8 (94.3, 99.4) • Convulsions, fits, or seizures: Sensitivity 15.0 (3.2, 37.9); Specificity 97.2 (93.5, 99.1) • Rapid breathing: Sensitivity 10.0 (1.2, 31.7); • Specificity 98.3 (95.1, 99.6) • Chest wall in-drawing: Sensitivity 0.0 (0.0, 16.1); Specificity 99.4 (96.9, 100) Validity of a combination of signs as an algorithm or index was not assessed.

Abbreviations: CBSV, community-based surveillance volunteers; CHW, community health worker; DiPS, district-based project supervisor; YICSS, Young Infants Clinical Signs Study.

### Development of the YICSS-Home Algorithm

#### Item generation

The development of the YICSS algorithm started with 31 infant signs on history and physical examination.^8^ These signs came from existing Integrated Management of Childhood Illness (IMCI) algorithms with some additional signs included to explore their use and predictive accuracy.^8^ According to the methods used to devise existing IMCI algorithms at the time, items were generated primarily by asking clinician experts on the study team.^31^

The list of 31 signs has some potentially important omissions including baseline risk factors for severe infant illness. These factors include maternal age, maternal education, infant sex, gestational age, birthweight and perinatal complications. The most important risk factors for neonatal sepsis are prematurity and low birthweight (<2500 g) with a 3 to 10 times higher incidence of infection in premature low birthweight infants compared to full-term normal birthweight infants.^32^ In LMICs, gestational age and birthweight are often difficult to obtain. Nevertheless, identification of preterm and low birthweight infants immediately after birth is recommended in WHO guidelines^5^ and should be encouraged. The addition of baseline risk factors for severe infant illness including low birthweight at the item generation stage would be important in future algorithm development.

#### Item reduction

A combination of a targeted statistical approach and judgmental approach was used to reduce items. For a predictive index used to identify risk, such as the YICSS-home algorithm, the target (criterion measure or gold standard) is the evidence of disease measured at the same time (concurrent) as the predictor variables.^33^ In the YICSS study, the target—severe illness warranting hospitalization as judged by a pediatrician—was measured within 2 hours of the measurement of the predictors (initial assessment of the infant by the primary health worker).^8^ This 2-hour interval is an important limitation since clinical signs may have changed during the interval. Importantly, the study pediatrician, supported by medical investigations, determining whether an infant had a serious illness warranting hospitalization (outcome) was blinded to the primary health worker’s findings (predictors).^8^

In the 0 to 6 day age group, a series of multiple logistic regression models was used to reduce items. The initial model included all signs that exhibited a univariate association with an odds ratio (OR) ≥2 and 95% CI that excluded 1. This model was then reduced from 31 items to 12 items by backward selection, excluding variables that did not meet predefined significance levels (OR < 2 or *P*-value > .05).^8^ This 12-sign algorithm requiring the presence of any one sign had a sensitivity of 87% and specificity of 74%. The algorithm was then further reduced to 7 signs based on clinical judgment, omitting signs with low prevalence.^8^ Of note, “jaundice” was not included as a clinical sign in the analysis used to generate the 7-sign YICSS algorithm because it was felt that although hyperbilirubinemia warranted admission to hospital, it was not a “severe illness.” “Any jaundice in first 24 hour of life, or yellow palms and soles at any age” was later added as the eighth sign to the YICSS-home algorithm based on a validation study.^28^ The 7-sign algorithm retained a sensitivity of 85% and specificity of 75%. The 7-sign algorithm was then applied to infants 7 to 59 days of age and had a sensitivity of 74% and specificity of 79% in this age group.

Each sign in the 7-sign algorithm carries equal weight. In the YICSS results, there was a wide range of ORs for each item, from OR of 2.7 for “respiratory rate ≥60” to OR of 15.4 for “history of convulsions.”^8^ Given this wide range of ORs, the items could be weighted to reflect the relative importance of each item based on ORs or regression coefficients. Moreover, the 7-sign algorithm was developed using the full cohort of 3177 infants aged 0 to 6 days and then applied to the 5712 infants aged 7 to 59 days. No internal validation process was used in item reduction when the algorithm was developed in the 0 to 6 days age group. Item reduction could therefore be improved by applying weights to items and performing internal validation.

### Sensibility

Sensibility is assessed using a combination of common sense and pathophysiological and clinical knowledge to qualitatively evaluate what a measurement tool contains and what it does.^24^ We evaluated the sensibility of the YICSS-home algorithm using Feinstein’s^24^ framework.

#### Purpose and framework

Feinstein^24^ states that every clinical index must have a purpose that is characterized by the clinical function it serves, the justification for its existence and its clinical applicability. The developers clearly specified in the YICSS study that the clinical function of the YICSS-home algorithm is to predict an outcome, in this case, severe illness warranting hospitalization as judged by a pediatrician.^8^ The developers provide clinical justification for the algorithm including (1) the need to identify young infants with severe illnesses by first-level health workers to reduce infant mortality in LMICs, and (2) to improve the previously existing algorithm^34^ that excluded infants in the first week of life.^8^

Data was collected from health facilities across multiple LMICs including Bangladesh, Bolivia, Ghana, India, Pakistan, and South Africa.^8^ The YICSS study included infants 0 to 59 days of age brought for care to a health facility due to caregiver concern. Thus, in terms of clinical applicability, the YICSS-home algorithm is applicable to infants presenting to a variety of LMIC health facility settings. However, the circumstance of caregivers actively seeking care for their infants greatly increases the pretest probability that clinical signs detected on assessment will predict a poor outcome such as hospitalization. Therefore, the findings of the YICSS study cannot be directly applied to screening approaches such as routine home visit infant assessments.

#### Comprehensibility

Comprehensibility can be defined by the principles of (1) simplicity (simple output scale), (2) oligovariability (minimal number of variables in the index), (3) transparency (minimal number of variables, categories in the rating scale used for each variable, and variation of weighting coefficients in an additive score), and (4) biologic connotation (challenge of associating a numerical score with a biologic connotation).^24^ Given the use of 2 categories in the binary scale (yes/no) for each item, the small number of variables (8), and the absence of weighting coefficients or additive score, the algorithm preserves Feinstein’s principles of simplicity, oligovariability and transparency. No score is generated that needs to be interpreted or associated with a biologic connotation. The output is simply that an infant has a severe illness warranting hospitalization if the infant has any one or more of the signs in the algorithm, which is easily comprehensible.

#### Replicability

Replicability refers to the clarity and thoroughness of the instructions provided and degree of biased examining (bias that may be introduced because of the attitudes or expectations of the person administering the index).^24^ Use of the YICSS-home algorithm is closely integrated with IMCI Chart Booklets and WHO training manuals for assessment of sick infants by CHWs.^35,36^ Clear instructions are provided on how to ask, look, listen and feel for the algorithm signs.^35,36^ The algorithm may be subject to biased examining. Length of CHW training varies widely, from 4 hours to 6 months.^37^ A CHW’s level of training and experience in recognizing the infant signs can affect the objectivity of administration of the algorithm.

#### Suitability of scale

The suitability of the output scale is determined by the comprehensiveness and discrimination of the scale.^24^ Regarding comprehensiveness, the binary scale of the YICSS-home algorithm has an exhaustive scope of categories (yes/no). The output scale also allows for easy discrimination. That is, the scale can be used to easily distinguish whether an infant has a severe illness warranting hospitalization both between different infants and within successive home visits for the same infant.

#### Face validity

Assessing face validity requires an appraisal of the (1) focus of interpersonal exchange (whether the person administering the index solicits information from the respondent in a manner that will evoke an accurate response), (2) focus of basic evidence (agreement between the purpose of the index and the phenomena described by the index), (3) biologic coherence of components (retention of coherence when aggregating multiple variables), and (4) attention to personal collaboration (attention given to collaboration between the person administering the index and the person to whom the index applies).^24^ CHWs live in the communities they serve and understand the history and context in which their patients live.^37^ They are therefore apt to provide culturally appropriate care, which supports the focus of interpersonal exchange.

The algorithm has an appropriate focus of basic evidence. That is, the purpose of the index and the type of evidence it contains are in agreement and the algorithm is directed at the correct target (severe illness requiring referral to hospital).

The biologic coherence of components is not a concern for this algorithm because there is no aggregation of multiple variables.

The signs in the algorithm are generally not affected by attention to personal collaboration except for “stopped feeding well.” In LMIC settings where infants are predominantly breastfed, male CHWs may be unable to observe and assess the quality of breastfeeding due to cultural sensitivity.^27^ “Stopped feeding well” should be assessed both by history and observation, but would often only be able to be assessed by history alone when the home visit is done by a male CHW. This limitation could be mitigated by clearly defining “stopped feeding well” as an item to be obtained on history only and re-evaluating its statistical significance as a history-only item.

#### Content validity

Content validity refers to the suitability of an index’s component parts including (1) omission of important variables, (2) inclusion of inappropriate variables, (3) weighting of variables, (4) satisfactory elemental scales (rating scales of variables), and (5) the quality of basic data (scientific quality of variables).^24^ Potentially important omissions in the YICSS-home algorithm include baseline maternal and birth history risk factors for severe infant illness such as low birthweight. Including low birthweight as a sign when developing the algorithm may have assigned more weight to certain variables. Moreover, when combined with low birthweight, signs that were omitted during item reduction (eg, “blood in stool”) may have been retained based on stronger statistical significance.

The only potentially inappropriate variable included in the YICSS-home algorithm is “stopped feeding well” given the potentially limited ability for this sign to be assessed by male CHWs in many LMIC settings. No weighting of variables was done. The elemental scale (yes/no) is satisfactory. The simplicity and coarseness of the scale is suitable for the algorithm’s purpose which is for a CHW to rapidly identify an infant with severe illness and refer for further evaluation. The variables consist of data gathered from history taking and physical examination. While the quality of the basic data would be better by also gathering data from laboratory tests, the exclusion of laboratory data is appropriate given the intended purpose and setting of the algorithm which is for use by CHWs during home visits in LMICs.

#### Ease of usage

Ease of usage refers to the amount of time, effort, and type of personnel needed to obtain the information used in the index.^24^ The YICSS-home algorithm is easy to use. It can be administered in a time-frame suitable for a home visit assessment. There are no special devices needed apart from a thermometer.

### Reliability

Reliability refers to the degree to which measurement is free from measurement error.^38^ An index is reliable when the same or close to the same measurement is obtained when repeated by the same rater or a different rater.

For the YICSS-home algorithm, the scale of each item is categorical (yes/no). For categorical measures, reliability is assessed using Cohen’s kappa statistic which is a measure of reliability that adjusts for the agreement that is expected by chance.^38^

Five studies assessed the algorithm’s inter-rater reliability by comparing agreement between a CHW (or CHW equivalent such as a community-based surveillance volunteer (CBSV) or female community health volunteer (FCHV)) and a more highly-trained health worker (district-based project supervisor [DiPS], facility-based community health worker [FB-CHW] or physician) (Table 2).^14,26-28,30^ In Darmstadt et al,^27^ kappas between CHWs and physicians for individual YICSS-home algorithm signs ranged from 0.08 to 0.67; in other studies, kappas ranged from 0.67 to 1.00 which represents substantial to almost perfect agreement according to the scale by Landis and Koch.^25^ For referral decisions,^14^ classification of “very severe disease,”^26^ and administration of the full YICSS-home algorithm (all 8 original signs),^28^ kappas ranged from 0.46 to 0.87 (moderate to almost perfect agreement).

To our knowledge, no study has assessed the inter-rater reliability of all 8 YICSS-home algorithm signs between CHWs or intra-rater reliability by assessing the measurements made by the same individual CHW on different occasions.

### Criterion Validity

Criterion validity is the degree to which scores of a measurement tool adequately reflect a gold standard or criterion measure.^38^ Concurrent criterion validity refers to how well the algorithm predicts the gold standard (severe illness warranting hospitalization) when both are measured at the same time. Predictive criterion validity refers to how well the algorithm predicts the gold standard at a later time.

Three studies assessed the criterion validity of the YICSS-home algorithm (Table 3).^14,28,29^ In Ansah Manu et al^14^ and Darmstadt et al,^28^ concurrent criterion validity was evaluated. The criterion used was an assessment performed by a DiPS Ansah Manu et al^14^ or a physician Darmstadt et al^28^ determining that a neonate had a severe illness requiring referral to hospital. Gill et al^29^ evaluated the predictive criterion validity of individual YICSS-home algorithm signs assessed by traditional birth attendants using death as the criterion. However, the validity of a combination of signs as an algorithm was not assessed.

Selecting a criterion for severe neonatal illness warranting hospitalization is particularly challenging because there is a wide range of illnesses in a neonate that may be considered to be severe and requiring hospitalization. In Darmstadt et al,^28^ there is no information provided about the validity or reliability of physicians’ judgment of need for hospital referral and the inter-observer reliability among physicians was not assessed. As such, it is difficult to evaluate the adequacy of the criterion. A more suitable criterion may have been to specifically use pediatricians’ judgment rather than general physicians’ judgment since pediatricians have more experience with infant illness.

An instrument needs evidence of validity in the target population and setting in which it will be used.^38^ Ansah Manu et al^14^ and Darmstadt et al^28^ assessed criterion validity of the YICSS-home algorithm among neonates (0-8 days) during home visits. Therefore, the study sample did not reflect the full target population in which the instrument will ultimately be used—infants up to 2 months of age. When assessing concurrent criterion validity, the scores of the index and the gold standard should be considered at the same time and should be obtained independently.^38^ In Ansah Manu et al,^14^ CBSVs and DiPS assessed neonates at the same time during directly observed supervisory visits. However, the DiPS were not blinded to the CBSVs’ assessments. In Darmstadt et al,^28^ physicians assessed neonates less than 12 hours after the CHWs’ assessments either at home (96%) or at the hospital (4%) and were appropriately blinded to the CHWs’ assessment results. On average, the time between CHW and physician assessment was 3 hours. This average lapse of 3 hours due to logistical reasons limits the results of the study since clinical signs may have changed during this period.

Furthermore, it is important to define a priori the required level of predictive accuracy between the instrument and the criterion.^38^ Darmstadt et al^28^ predefined a sensitivity of 70% and specificity of 80%. In this study, the YICSS-home algorithm used by CHWs had a sensitivity of 68.8% (95% CI 41.3%, 89.0%) and specificity of 95.3% (95% CI 92.6%, 97.2%) compared to physician assessment. In Ansah Manu et al,^14^ no level of accuracy was predefined. An 8-sign algorithm, that included 6 of the 8 YICSS-home algorithm signs, used by CBSVs identified neonates requiring referral with a sensitivity of 79.5% and specificity of 100% compared to DiPS assessment. The discrepancy between the sensitivity and specificity of the YICSS-home algorithm used in Darmstadt et al^28^ and the algorithm used in Ansah Manu et al^14^ may have been due to the differences in 2 of the signs. For a screening tool, a sensitivity of 80% and specificity of 90% have been recommended.^39^ Low sensitivity may lead to under-referral and missing cases of severe infant illness.

### Construct Validity

Construct validity is the degree to which scores of a measurement tool are consistent with theoretical a priori defined hypotheses regarding internal relationships, relationships with scores of other tools or differences between relevant groups.^38^ Basic construct validity should be established.^40^ No study has assessed the construct validity of the YICSS-home algorithm.

## Discussion

This critical appraisal of the YICSS-home algorithm demonstrated some strengths and several important limitations. The YICSS-home algorithm is replicable, comprehensible, simple and can be administered in a short time-frame suitable for a home visit assessment. Given that CHWs live in the communities they serve, they are apt to provide culturally appropriate care. The simplicity of the algorithm is particularly important given that CHWs have minimal medical training and it would therefore be costly and challenging to train CHWs to learn and apply an algorithm involving many items or one that is medically complex. No special devices are needed apart from a thermometer, and no laboratory data are required which makes the algorithm practical for use by CHWs during home visits in LMICs. In the algorithm’s development and criterion validation, the study pediatrician determining whether an infant had a severe illness warranting hospitalization (outcome) was appropriately blinded to the CHW’s assessment (predictors). Reliability was mostly moderate to almost perfect between CHWs and a more highly-trained health worker for both assessment of individual signs and classification of severe illness.

The most significant limitation of the YICSS-home algorithm is that it was originally developed using a cohort of infants brought for care to a health facility due to caregiver concern and not initially developed for use by CHWs in the home visit setting. Other important limitations include omissions at item generation of maternal and birth history risk factors for severe infant illness, namely low birthweight. Weighting of items and internal validation may have improved the algorithm’s performance. The item “stopped feeding well” may not be directly observable by male CHWs due to cultural sensitivity in LMICs. Inter- and intra-CHW reliability and construct validity have not been assessed. Assessment of concurrent criterion validity of the YICSS-home algorithm demonstrated sensitivity ranging from 69% to 80%, raising potential concern for under-referral of infants and missing cases of severe illness.

Future research should build on the strengths of the YICSS-home algorithm and address its limitations to develop a new algorithm with improved predictive accuracy. When developing a new algorithm, we recommend: (1) using a cohort of infants assessed by CHWs during home visits; (2) including maternal and birth history risk factors at the item generation stage or adjusting for these risk factors in the analysis; (3) performing internal validation; and (4) estimating inter- and intra-CHW reliability, criterion validity and construct validity.

Assessing the inter- and intra-CHW reliability of the YICSS-home algorithm may be practically and ethically challenging. For example, it may not be ethical for a potentially severely ill infant to be assessed multiple times by different CHWs for research purposes before receiving appropriate care. A more ethically appropriate study could involve CHW assessment of a sample of videos of infants exhibiting the YICSS-home algorithm signs in home settings in LMICs.

A study to evaluate the construct validity of the YICSS-home algorithm for measuring severe infant illness could be done using known groups construct validity. Evaluating known groups construct validity involves identifying known groups and formulating hypotheses about expected differences between groups. For example, it is known that low birthweight infants (<2500 g) have a higher risk of poor outcomes such as hospitalization or mortality than normal birthweight infants (≥2500 g).^32^ Using these known groups, we could hypothesize that infants with low birthweight are more likely to experience the outcome of severe illness than infants with normal birthweight. The construct to be measured would be severe illness in infants warranting hospitalization as judged by a pediatrician. The hypothesis would be tested by having CHWs apply the YICSS-home algorithm to these 2 different infant populations (low birthweight vs normal birthweight) during home visits and assessing the difference in scores using logistic regression.

Developing an algorithm among infants assessed in the home visit setting offers the possibility of harnessing repeated measurements of predictors over multiple home visits. Emerging evidence suggests improved predictive accuracy for prediction models that use repeated measurements compared to traditional prediction models that use single measurements.^41-43^ In the home visit setting, repeated measurements of clinical signs ascertained from sequential home visits^7,44^ can be utilized in terms of their recurrence and combinations. For example, let us say that a CHW determined that an infant had “severe chest in-drawing” on day 10 of an evolving pneumonia and, using the YICSS-home algorithm, s/he referred the infant to a hospital on day 10. However, recurrence and combinations of other milder clinical features (eg, cough, runny nose, temperature of 37.4°C) may have been detected during home visits prior to day 10 of illness. These features could potentially have been utilized to predict the pneumonia earlier than day 10. Earlier detection of illness could prompt earlier intervention such as closer follow-up of the infant.

Furthermore, features from the YICSS-home algorithm defined using general thresholds such as “low body temperature (<35.5°C)” may have poor predictive accuracy for severe illness because infants’ normal temperature ranges may vary depending on the setting. One systematic review investigating the global burden of neonatal hypothermia found that the prevalence of hypothermia varied depending on environmental temperatures.^45^ Developing a new algorithm in the home visit setting allows for an infant’s temperature to be compared to prior measurements rather than general thresholds. For example, an item could be defined as “2 standard deviations below the infant’s mean temperature based on prior home visit measurements.” A new algorithm that includes such an item may be more accurate and widely applicable than the use of a general threshold for “low body temperature” in the YICSS-home algorithm.

This review has several limitations. First, our review was limited by the availability of the evidence since there were no studies reporting inter- or intra-CHW reliability, or construct validity. Second, we did not formally assess the risk of bias of each included study. However, we have critically appraised the quality of the development of the YICSS-home algorithm and available evidence of its reliability and criterion validity for its intended use, which informs the interpretation of the results of included studies. Finally, no authors from LMICs were included in the conduct of this review. Future research should encourage representation from LMIC co-authors including CHWs who use these algorithms in practice.

## Conclusions

### Implications for Policy, Practice and Research

The YICSS-home algorithm demonstrates good sensibility, moderate to almost perfect inter-rater reliability, and is a practical tool to support the identification of sick infants requiring referral during CHW home visits. However, the algorithm has several important limitations. Future research should build on the strengths of the YICSS-home algorithm and address its limitations to develop a new algorithm with improved predictive accuracy. When developing a new algorithm, we recommend using a cohort of infants assessed by CHWs during home visits, including maternal and birth history risk factors, performing internal validation, and estimating inter- and intra-CHW reliability, criterion validity and construct validity. Incorporation of repeated measurements of clinical signs from multiple home visits may be explored to potentially improve predictive accuracy.

## Supplemental Material

sj-docx-1-gph-10.1177_2333794X231219598 – Supplemental material for Young Infants Clinical Signs Study 8-sign Algorithm for Identification of Sick Infants Adapted for Routine Home Visits: A Systematic Review and Critical Appraisal of its Measurement Properties Click here for additional data file. Supplemental material, sj-docx-1-gph-10.1177_2333794X231219598 for Young Infants Clinical Signs Study 8-sign Algorithm for Identification of Sick Infants Adapted for Routine Home Visits: A Systematic Review and Critical Appraisal of its Measurement Properties by Alastair Fung, Julie Farmer and Cornelia M. Borkhoff in Global Pediatric Health

sj-docx-2-gph-10.1177_2333794X231219598 – Supplemental material for Young Infants Clinical Signs Study 8-sign Algorithm for Identification of Sick Infants Adapted for Routine Home Visits: A Systematic Review and Critical Appraisal of its Measurement Properties Click here for additional data file. Supplemental material, sj-docx-2-gph-10.1177_2333794X231219598 for Young Infants Clinical Signs Study 8-sign Algorithm for Identification of Sick Infants Adapted for Routine Home Visits: A Systematic Review and Critical Appraisal of its Measurement Properties by Alastair Fung, Julie Farmer and Cornelia M. Borkhoff in Global Pediatric Health

sj-docx-3-gph-10.1177_2333794X231219598 – Supplemental material for Young Infants Clinical Signs Study 8-sign Algorithm for Identification of Sick Infants Adapted for Routine Home Visits: A Systematic Review and Critical Appraisal of its Measurement Properties Click here for additional data file. Supplemental material, sj-docx-3-gph-10.1177_2333794X231219598 for Young Infants Clinical Signs Study 8-sign Algorithm for Identification of Sick Infants Adapted for Routine Home Visits: A Systematic Review and Critical Appraisal of its Measurement Properties by Alastair Fung, Julie Farmer and Cornelia M. Borkhoff in Global Pediatric Health
