# Supplementary material for: Young Infants Clinical Signs Study 8-sign Algorithm for Identification of Sick Infants Adapted for Routine Home Visits: A Systematic Review and Critical Appraisal of its Measurement Properties
Source: Glob Pediatr Health. 2024 Jan 25;11:2333794X231219598. doi: 10.1177/2333794X231219598 (PMC10812101; doi:10.1177/2333794X231219598)
Supplement: sj-docx-1-gph-10.1177_2333794X231219598 – Supplemental material for Young Infants Clinical Signs Study 8-sign Algorithm for Identification of Sick Infants Adapted for Routine Home Visits: A Systematic Review and Critical Appraisal of its Measurement Properties [file sj-docx-1-gph-10.1177_2333794X231219598.docx]

**Supplemental Table 3.** Reasons for exclusion at full-text review

| Author Year | Title | Reason for exclusion |
| --- | --- | --- |
| Anonymous 1999 | Conclusions from the WHO multicenter study of serious infections in young infants. The WHO Young Infants Study Group. | Assessor not a CHW |
| Anonymous 1999 | Clinical prediction of serious bacterial infections in young infants in developing countries. The WHO Young Infants Study Group. | Assessor not a CHW |
| Bandyopadhyay et al. 2003 | Are primary health workers skilled enough to assess the severity of illness among young infants? | Setting not relevant |
| Bang et al. 2005 | Simple clinical criteria to identify sepsis or pneumonia in neonates in the community needing treatment or referral. | Fewer than 5 of the 8 YICSS-home algorithm signs |
| Coghill et al. 2011 | Which clinical signs predict severe illness in children less than 2 months of age in resource poor countries? | Review article |
| Deorari et al. 2007 | Clinicoepidemiological profile and predictors of severe illness in young infants (< 60 days) reporting to a hospital in North India. | Setting not relevant |
| Getachew et al. 2019 | Health Extension Workers' diagnostic accuracy for common childhood illnesses in four regions of Ethiopia: a cross-sectional study. | Population not relevant |
| Hansoti et al. 2020 | Prioritizing the Care of Critically Ill Children in South Africa: How Does SCREEN Perform Against Other Triage Tools?. | Setting not relevant |
| Harrell et al. 1998 | Development of a clinical prediction model for an ordinal outcome: the World Health Organization Multicentre Study of Clinical Signs and Etiological agents of Pneumonia, Sepsis and Meningitis in Young Infants. WHO/ARI Young Infant Multicentre Study Group. | Assessor not a CHW |
| Lunze et al. 2017 | Clinical management of children with fever: A cross-sectional study of quality of care in rural Zambia | Setting not relevant |
| Narang et al. 2007 | Clinico-epidemiological profile and validation of symptoms and signs of severe illness in young infants (< 60 days) reporting to a district hospital. | Setting not relevant |
| Paxton et al. 1996 | An evaluation of clinical indicators for severe paediatric illness. | Setting not relevant |
| Shahid et al. 2021 | Clinical signs predictive of severe illness in young Pakistani infants. | Setting not relevant |
| Shewade et al. 2013 | Integrated Management of Neonatal and Childhood Illness (IMNCI): skill assessment of health and Integrated Child Development Scheme (ICDS) workers to classify sick under-five children. | Setting not relevant |
| Weber et al. 2003 | Predictors of neonatal sepsis in developing countries | Assessor not a CHW |
