# Supplementary material for: Young Infants Clinical Signs Study 8-sign Algorithm for Identification of Sick Infants Adapted for Routine Home Visits: A Systematic Review and Critical Appraisal of its Measurement Properties
Source: Glob Pediatr Health. 2024 Jan 25;11:2333794X231219598. doi: 10.1177/2333794X231219598 (PMC10812101; doi:10.1177/2333794X231219598)
Supplement: sj-docx-2-gph-10.1177_2333794X231219598 – Supplemental material for Young Infants Clinical Signs Study 8-sign Algorithm for Identification of Sick Infants Adapted for Routine Home Visits: A Systematic Review and Critical Appraisal of its Measurement Properties [file sj-docx-2-gph-10.1177_2333794X231219598.docx]

**Supplemental Table 2.** Search strategies

Database(s): **Ovid MEDLINE: Epub Ahead of Print, In-Process & Other Non-Indexed Citations, Ovid MEDLINE® Daily and Ovid MEDLINE®**1946-Present
Search Strategy:

| **#** | **Searches** | **Results** |
| --- | --- | --- |
| 1 | exp Infant/ or exp Newborn/ | 1223258 |
| 2 | (("infant" and "newborn") or "newborn infant" or "neonatal" or (neonat* or newborn*)).mp. [mp=title, abstract, original title, name of substance word, subject heading word, floating sub-heading word, keyword heading word, organism supplementary concept word, protocol supplementary concept word, rare disease supplementary concept word, unique identifier, synonyms] | 910090 |
| 3 | 1 or 2 | 1468422 |
| 4 | Community Health Workers/ | 6285 |
| 5 | "community health worker*".tw. | 5402 |
| 6 | "primary health worker*".tw. | 140 |
| 7 | "health volunteer*".mp. | 1039 |
| 8 | "health worker*".mp. | 25316 |
| 9 | "front?line".mp. | 11517 |
| 10 | Nursing Assessment/ | 29079 |
| 11 | exp Neonatal Screening/ | 11489 |
| 12 | "neonatal screening".tw. | 3150 |
| 13 | "Integrated Management of Childhood Illness*".tw. | 491 |
| 14 | "IMCI".tw. | 404 |
| 15 | Integrated Management of Newborn.mp. and Childhood Illness*.tw. [mp=title, abstract, original title, name of substance word, subject heading word, floating sub-heading word, keyword heading word, organism supplementary concept word, protocol supplementary concept word, rare disease supplementary concept word, unique identifier, synonyms] | 8 |
| 16 | "IMNCI".tw. | 58 |
| 17 | "danger sign*".tw. | 3979 |
| 18 | "Young Infant* Study".tw. | 7 |
| 19 | "Young Infant* Sign*".tw. | 4 |
| 20 | "Young Infant* Clinical Sign*".tw. | 1 |
| 21 | "Young Infant* Clinical Sign* Study".mp. | 0 |
| 22 | "algorithm*".mp. | 477901 |
| 23 | exp Child Health Services/ | 25475 |
| 24 | Risk Assessment/ | 300379 |
| 25 | "risk assessment".mp. | 345401 |
| 26 | "clinical decision*".mp. | 53854 |
| 27 | 4 or 5 or 6 or 7 or 8 or 9 or 10 or 11 or 12 or 13 or 14 or 15 or 16 or 17 or 18 or 19 or 20 or 21 or 22 or 23 or 24 or 25 or 26 | 961816 |
| 28 | ("illness" or "disease" or "survival").tw. | 4503878 |
| 29 | "infection".mp. | 1362180 |
| 30 | infections/ or "bacterial infections and mycoses"/ | 40568 |
| 31 | ("septicemia" or "sepsis").mp. or exp sepsis/ or exp Neonatal Sepsis/ | 208229 |
| 32 | ("possible serious bacterial infection" or "possible severe bacterial infection" or "PSBI" or "serious bacterial infection" or "SBI").tw. | 2269 |
| 33 | ("serious illness*" or "severe illness*" or "severe disease*" or "serious disease*").tw. | 42154 |
| 34 | "sick infant*".tw. | 544 |
| 35 | "sick young infant*".tw. | 38 |
| 36 | "sick newborn*".tw. | 575 |
| 37 | "sick neonate*".tw. | 553 |
| 38 | "mortality".tw. | 898105 |
| 39 | exp Communicable Diseases/ | 524329 |
| 40 | "communicable disease".tw. | 4464 |
| 41 | ("communicable" adj3 "disease*").tw. | 16001 |
| 42 | bacteremia/ or "bacter?emia".mp. | 46126 |
| 43 | meningitis.mp. or exp Meningitis/ | 80536 |
| 44 | "urinary tract infection*".mp. or exp Urinary Tract Infections/ | 71561 |
| 45 | ("urinary tract" adj3 "infection*").mp. | 65344 |
| 46 | Pneumonia/ or Pneumonia.mp. | 216611 |
| 47 | ("acute" and "lower" and "respiratory tract infection*").mp. | 3981 |
| 48 | Respiratory Tract Infections/ | 41650 |
| 49 | ("respiratory" and "tract" and "infection*").mp. | 73617 |
| 50 | ("respiratory" adj3 "infection*").mp. [mp=title, abstract, original title, name of substance word, subject heading word, floating sub-heading word, keyword heading word, organism supplementary concept word, protocol supplementary concept word, rare disease supplementary concept word, unique identifier, synonyms] | 86434 |
| 51 | Hospitalization/ and Humans/ | 126694 |
| 52 | hospitalization/ or patient admission/ | 152349 |
| 53 | "hospitali#ation*".mp. | 278100 |
| 54 | ("hospitali#*" or "admit*" or "admission*").tw. | 682536 |
| 55 | or/28-54 | 6862666 |
| 56 | (instrumentation or methods).sh. | 231752 |
| 57 | (validation study or comparative study).pt. | 2000060 |
| 58 | exp Psychometrics/ | 85154 |
| 59 | psychometr*.tw. | 55748 |
| 60 | (clinimetr* or clinometr*).mp. | 1439 |
| 61 | exp Outcome Assessment, Health Care/ | 1292301 |
| 62 | outcome assessment.tw. | 4594 |
| 63 | outcome measure*.mp. | 270172 |
| 64 | exp Observer Variation/ | 44661 |
| 65 | observer variation.tw. | 1161 |
| 66 | exp Health Status Indicators/ | 337877 |
| 67 | exp Reproducibility of Results/ | 450146 |
| 68 | reproducib*.tw. | 180679 |
| 69 | exp Discriminant Analysis/ | 11724 |
| 70 | (reliab* or unreliab* or valid* or coefficient of variation or coefficient or homogeneity or homogeneous or internal consistency).tw. | 1672579 |
| 71 | (cronbach* and (alpha or alphas)).tw. | 28638 |
| 72 | (item and (correlation* or selection* or reduction*)).tw. | 27390 |
| 73 | agreement.mp. | 305153 |
| 74 | precision.mp. | 183265 |
| 75 | imprecision.mp. | 7285 |
| 76 | precise values.mp. | 304 |
| 77 | test-retest.tw. | 30569 |
| 78 | (test and retest).tw. | 32004 |
| 79 | (reliab* and (test or retest)).tw. | 105501 |
| 80 | stability.tw. | 509753 |
| 81 | (interrater or inter-rater or intrarater or intra-rater).tw. | 23891 |
| 82 | (intertester or inter-tester or intratester or intra-tester).tw. | 667 |
| 83 | (interobserver or inter-observer or intraobserver or intra-observer).tw. | 32288 |
| 84 | (intertechnician or inter-technician or intratechnician or intra-technician).tw. | 22 |
| 85 | (interexaminer or inter-examiner or intraexaminer or intra-examiner).tw. | 2286 |
| 86 | (interassay or inter-assay or intraassay or intra-assay).tw. | 9739 |
| 87 | (interindividual or inter-individual or intraindividual or intra-individual).tw. | 39364 |
| 88 | (interparticipant or inter-participant or intraparticipant or intra-participant).tw. | 191 |
| 89 | kappa.tw. | 105359 |
| 90 | kappas.tw. | 1161 |
| 91 | repeatab*.mp. | 41576 |
| 92 | ((replicab* or repeated) and (measure or measures or findings or result or results or test or tests)).mp. | 228689 |
| 93 | (generaliza* or generalisa*).tw. | 55716 |
| 94 | concordance.tw. | 54138 |
| 95 | (intraclass and correlation*).tw. | 31113 |
| 96 | discriminative.tw. | 22553 |
| 97 | known group.tw. | 1383 |
| 98 | (factor analysis or factor analyses or factor structure or factor structures).tw. | 62627 |
| 99 | dimension*.tw. | 661053 |
| 100 | subscale*.tw. | 52085 |
| 101 | (multitrait and scaling and (analysis or analyses)).tw. | 150 |
| 102 | item discriminant.tw. | 122 |
| 103 | interscale correlation*.tw. | 166 |
| 104 | (error or errors).tw. | 350386 |
| 105 | individual variability.tw. | 10081 |
| 106 | interval variability.tw. | 684 |
| 107 | rate variability.tw. | 21602 |
| 108 | (variability and (analysis or values)).tw. | 115143 |
| 109 | (uncertainty and (measurement or measuring)).tw. | 9409 |
| 110 | standard error of measurement.tw. | 2397 |
| 111 | sensitiv*.tw. | 1558683 |
| 112 | responsive*.tw. | 258756 |
| 113 | (limit and detection).tw. | 108400 |
| 114 | minimal detectable concentration.tw. | 85 |
| 115 | interpretab*.tw. | 13634 |
| 116 | ((minimal or minimally or clinical or clinically) and (important or significant or detectable) and (change or difference)).tw. | 282054 |
| 117 | (small* and (real or detectable) and (change or difference)).tw. | 8763 |
| 118 | meaningful change.tw. | 1310 |
| 119 | ceiling effect.tw. | 2144 |
| 120 | floor effect.tw. | 748 |
| 121 | item response model.tw. | 156 |
| 122 | IRT.tw. | 3894 |
| 123 | rasch.tw. | 4999 |
| 124 | differential item functioning.tw. | 2155 |
| 125 | DIF.tw. | 3147 |
| 126 | computer adaptive testing.tw. | 239 |
| 127 | item bank.tw. | 722 |
| 128 | cross-cultural equivalence.tw. | 138 |
| 129 | or/56-128 | 8460606 |
| 130 | (afghanistan or albania or algeria or american samoa or angola or "antigua and barbuda" or antigua or barbuda or argentina or armenia or armenian or aruba or azerbaijan or bahrain or bangladesh or barbados or republic of belarus or belarus or byelarus or belorussia or byelorussian or belize or british honduras or benin or dahomey or bhutan or bolivia or "bosnia and herzegovina" or bosnia or herzegovina or botswana or bechuanaland or brazil or brasil or bulgaria or burkina faso or burkina fasso or upper volta or burundi or urundi or cabo verde or cape verde or cambodia or kampuchea or khmer republic or cameroon or cameron or cameroun or central african republic or ubangi shari or chad or chile or china or colombia or comoros or comoro islands or iles comores or mayotte or democratic republic of the congo or democratic republic congo or congo or zaire or costa rica or "cote d’ivoire" or "cote d’ ivoire" or cote divoire or cote d ivoire or ivory coast or croatia or cuba or cyprus or czech republic or czechoslovakia or djibouti or french somaliland or dominica or dominican republic or ecuador or egypt or united arab republic or el salvador or equatorial guinea or spanish guinea or eritrea or estonia or eswatini or swaziland or ethiopia or fiji or gabon or gabonese republic or gambia or "georgia (republic)" or georgian or ghana or gold coast or gibraltar or greece or grenada or guam or guatemala or guinea or guinea bissau or guyana or british guiana or haiti or hispaniola or honduras or hungary or india or indonesia or timor or iran or iraq or isle of man or jamaica or jordan or kazakhstan or kazakh or kenya or "democratic people’s republic of korea" or republic of korea or north korea or south korea or korea or kosovo or kyrgyzstan or kirghizia or kirgizstan or kyrgyz republic or kirghiz or laos or lao pdr or "lao people's democratic republic" or latvia or lebanon or lebanese republic or lesotho or basutoland or liberia or libya or libyan arab jamahiriya or lithuania or macau or macao or republic of north macedonia or macedonia or madagascar or malagasy republic or malawi or nyasaland or malaysia or malay federation or malaya federation or maldives or indian ocean islands or indian ocean or mali or malta or micronesia or federated states of micronesia or kiribati or marshall islands or nauru or northern mariana islands or palau or tuvalu or mauritania or mauritius or mexico or moldova or moldovian or mongolia or montenegro or morocco or ifni or mozambique or portuguese east africa or myanmar or burma or namibia or nepal or netherlands antilles or nicaragua or niger or nigeria or oman or muscat or pakistan or panama or papua new guinea or new guinea or paraguay or peru or philippines or philipines or phillipines or phillippines or poland or "polish people's republic" or portugal or portuguese republic or puerto rico or romania or russia or russian federation or ussr or soviet union or union of soviet socialist republics or rwanda or ruanda or samoa or pacific islands or polynesia or samoan islands or navigator island or navigator islands or "sao tome and principe" or saudi arabia or senegal or serbia or seychelles or sierra leone or slovakia or slovak republic or slovenia or melanesia or solomon island or solomon islands or norfolk island or norfolk islands or somalia or south africa or south sudan or sri lanka or ceylon or "saint kitts and nevis" or "st. kitts and nevis" or saint lucia or "st. lucia" or "saint vincent and the grenadines" or saint vincent or "st. vincent" or grenadines or sudan or suriname or surinam or dutch guiana or netherlands guiana or syria or syrian arab republic or tajikistan or tadjikistan or tadzhikistan or tadzhik or tanzania or tanganyika or thailand or siam or timor leste or east timor or togo or togolese republic or tonga or "trinidad and tobago" or trinidad or tobago or tunisia or turkey or turkmenistan or turkmen or uganda or ukraine or uruguay or uzbekistan or uzbek or vanuatu or new hebrides or venezuela or vietnam or viet nam or middle east or west bank or gaza or palestine or yemen or yugoslavia or zambia or zimbabwe or northern rhodesia or global south or africa south of the sahara or sub-saharan africa or subsaharan africa or africa, central or central africa or africa, northern or north africa or northern africa or magreb or maghrib or sahara or africa, southern or southern africa or africa, eastern or east africa or eastern africa or africa, western or west africa or western africa or west indies or indian ocean islands or caribbean or central america or latin america or "south and central america" or south america or asia, central or central asia or asia, northern or north asia or northern asia or asia, southeastern or southeastern asia or south eastern asia or southeast asia or south east asia or asia, western or western asia or europe, eastern or east europe or eastern europe or developing country or developing countries or developing nation? or developing population? or developing world or less developed countr* or less developed nation? or less developed population? or less developed world or lesser developed countr* or lesser developed nation? or lesser developed population? or lesser developed world or under developed countr* or under developed nation? or under developed population? or under developed world or underdeveloped countr* or underdeveloped nation? or underdeveloped population? or underdeveloped world or middle income countr* or middle income nation? or middle income population? or low income countr* or low income nation? or low income population? or lower income countr* or lower income nation? or lower income population? or underserved countr* or underserved nation? or underserved population? or underserved world or under served countr* or under served nation? or under served population? or under served world or deprived countr* or deprived nation? or deprived population? or deprived world or poor countr* or poor nation? or poor population? or poor world or poorer countr* or poorer nation? or poorer population? or poorer world or developing econom* or less developed econom* or lesser developed econom* or under developed econom* or underdeveloped econom* or middle income econom* or low income econom* or lower income econom* or low gdp or low gnp or low gross domestic or low gross national or lower gdp or lower gnp or lower gross domestic or lower gross national or lmic or lmics or third world or lami countr* or transitional countr* or emerging economies or emerging nation?).ti,ab,sh,kf. | 2237976 |
| 131 | (afghan or afghans or afghani or albanian? algerian? or american samoan? or angolan? or antiguan? or barbudan? or argentine? or argentinian? or argentinean? or armenian? or aruban? or azerbaijani? or bahraini? or bangladeshi? or bangalees or bajan? or belarusian? or byelorussian? or belizean? or beninese? or bhutanese or bolivian? or bosnian? or botswana or batswana or brazilian? or brasilian? or bulgarian? or burkinabe or burkinese or burundian? or cape verdean? or cabo verdean? or cambodian? or khmer or cameroonian? or central african? or chadian? or chilean? or chinese or colombian? or comorian? or congolese or costa rican? or ivorian? or croatian? or cuban? or cypriot? or czech? or djiboutian? or dominican? or ecuadorian? or egyptian? or salvadoran? or equatorial guinean? or equatoguinean? or eritrean? or estonian? or swazi? or swati? or ethiopian? or fijian or gabonese or gabonaise or gambian? or georgian? or ghanaian? or gibraltarian? or greek? or grenadian? or guamanian? or guatemalan? or guinean? or bissau guinean? or guyanese or haitian? or honduran? or hungarian? or indian? or indonesian? or iranian? or iraqian? or iraqi? or manx or jamaican? or jordanian? or kazakhstani? or kenyan? or kirabati or kirabatian? or north korean? or korean? or kosovar? or kosovan? or kyrgyz* or lao or laotian? or latvian? or lebanese or lesothan? or lesothonian? or mosotho or basotho or liberian? or libyan? or lithuanian? or macanese or macedonian? or malagasy or madagascan? or malawian? or malaysian? or maldivian? or malian? or maltese or marshallese? or mauritanian? or mauritian? or mexican? or micronesian? or moldovan? or mongolian? or mongol or montenegrin? or moroccan? or mozambican? or burmese or myanma or namibian? or nauruan? or nepali or nepalese or netherlands antillean? or nicaraguan? or nigerien? or nigerian? or northern mariana islander? or mariana? or omani? or pakistani? or palauan? or panamanian? or papua new guinean? or paraguayan? or peruvian? or philippine? or philipine? or phillipine? or phillippine? or filipino? or filipina? or polish or pole or poles or portuguese or puerto rican? or romanian? or russian? or soviet people or soviet population or rwandan? or rwandese or ruandan? or ruandese or samoan? or sao tomean? or santomean? or saudi arabian? or saudi? or senegalese or serbian? or montenegrin? or seychellois or seychelloise? or sierra leonean? or slovak? or slovene? or solomon islander? or somali? or south african? or south sudanese or sri lankan? or ceylonese or kittitian? or nevisian? or saint lucian? or vincentian? or sudanese or surinamese? or syrian? or tajik? or tajikistani? or tanzanian? or tanganyikan? or thai or timorese? or togolese or tongan? or trinidadian? or tobagonian? or tunisian? or turk? or turkish or turkmen? or tuvaluan? or ugandan? or ukrainian? or uruguayan? or uzbek? or vanuatu* or venezuelan? or vietnamese or yemeni? or yemenite? or yemenese or yugoslav? or yugoslavian? or zambian? or zimbabwean?).ti,ab,sh,kf. | 991702 |
| 132 | 130 or 131 | 2656295 |
| 133 | 3 and 27 and 55 | 22771 |
| 134 | 3 and 27 and 55 and 129 | 10488 |
| 135 | 3 and 27 and 55 and 129 and 132 | 2567 |

|  |  |
| --- | --- |

Database(s): **Embase Classic+Embase**1947 to 2022 July 12
Search Strategy:

| **#** | **Searches** | **Results** |
| --- | --- | --- |
| 1 | infant/ or baby/ or newborn/ | 1256031 |
| 2 | (("infant" and "newborn") or "newborn infant" or "neonatal" or (neonat* or newborn*)).mp. | 942994 |
| 3 | 1 or 2 | 1482796 |
| 4 | health auxiliary/ | 8785 |
| 5 | "community health worker*".tw. | 6648 |
| 6 | "primary health worker*".tw. | 148 |
| 7 | "health volunteer*".mp. | 1440 |
| 8 | "health worker*".mp. | 27032 |
| 9 | "front?line".mp. | 17930 |
| 10 | nursing assessment/ | 27412 |
| 11 | newborn screening/ | 21437 |
| 12 | "neonatal screening".tw. | 4702 |
| 13 | "Integrated Management of Childhood Illness*".tw. | 594 |
| 14 | "IMCI".tw. | 524 |
| 15 | "Integrated Management of Newborn and Childhood Illness*".mp. | 12 |
| 16 | "IMNCI".tw. | 78 |
| 17 | "danger sign*".tw. | 5685 |
| 18 | "Young Infant* Study".tw. | 11 |
| 19 | "Young Infant* Sign*".tw. | 4 |
| 20 | "Young Infant* Clinical Sign*".tw. | 2 |
| 21 | "Young Infant* Clinical Sign* Study".mp. | 1 |
| 22 | "algorithm*".mp. | 530859 |
| 23 | child health care/ or newborn care/ | 53721 |
| 24 | risk assessment/ or health risk assessment/ | 673336 |
| 25 | "risk assessment".mp. | 709067 |
| 26 | "clinical decision*".mp. | 100957 |
| 27 | or/4-26 | 1458117 |
| 28 | ("illness" or "disease" or "survival").tw. | 6608643 |
| 29 | "infection".mp. | 2827460 |
| 30 | infections/ or "bacterial infections and mycoses"/ | 406934 |
| 31 | sepsis/ or newborn sepsis/ or septicemia/ | 218501 |
| 32 | ("septicemia" or "sepsis").mp. | 283108 |
| 33 | ("possible serious bacterial infection" or "possible severe bacterial infection" or "PSBI" or "serious bacterial infection" or "SBI").tw. | 3318 |
| 34 | ("serious illness*" or "severe illness*" or "severe disease*" or "serious disease*").tw. | 62460 |
| 35 | "sick infant*".tw. | 738 |
| 36 | "sick young infant*".tw. | 42 |
| 37 | "sick newborn*".tw. | 753 |
| 38 | "sick neonate*".tw. | 783 |
| 39 | "mortality".tw. | 1363018 |
| 40 | communicable disease/ | 36030 |
| 41 | "communicable disease".tw. | 5363 |
| 42 | ("communicable" adj3 "disease*").tw. | 19529 |
| 43 | bacteremia/ or "bacter?emia".mp. | 66741 |
| 44 | meningitis.mp. or exp Meningitis/ | 148534 |
| 45 | "urinary tract infection*".mp. or exp Urinary Tract Infections/ | 153573 |
| 46 | ("urinary tract" adj3 "infection*").mp. | 141499 |
| 47 | Pneumonia/ or Pneumonia.mp. | 399309 |
| 48 | ("acute" and "lower" and "respiratory tract infection*").mp. | 8425 |
| 49 | Respiratory Tract Infection/ | 70877 |
| 50 | ("respiratory" and "tract" and "infection*").mp. | 175884 |
| 51 | ("respiratory" adj3 "infection*").mp. | 158818 |
| 52 | (hospitalization/ or child hospitalization/) and human/ | 456817 |
| 53 | hospitalization/ or hospital admission/ | 684421 |
| 54 | "hospitali#ation*".mp. | 601981 |
| 55 | ("hospitali#*" or "admit*" or "admission*").tw. | 1166463 |
| 56 | newborn disease/ | 35466 |
| 57 | or/28-56 | 10272047 |
| 58 | (instrumentation or methods).mp. | 4102326 |
| 59 | ("validation study" or "comparative study").mp. | 1146450 |
| 60 | exp Psychometrics/ | 107990 |
| 61 | psychometr*.tw. | 67913 |
| 62 | (clinimetr* or clinometr*).mp. | 2138 |
| 63 | exp Outcome Assessment, Health Care/ | 715681 |
| 64 | "outcome assessment".tw. | 6290 |
| 65 | "outcome measure*".mp. | 338895 |
| 66 | exp "Observer Variation"/ | 20747 |
| 67 | "observer variation".tw. | 1787 |
| 68 | exp "Health Status Indicators"/ | 38415 |
| 69 | exp "Reproducibility of Results"/ | 246712 |
| 70 | reproducib*.tw. | 237108 |
| 71 | exp "Discriminant Analysis"/ | 24170 |
| 72 | (reliab* or unreliab* or valid* or "coefficient of variation" or coefficient or homogeneity or homogenous or "internal consistency").tw. | 2162527 |
| 73 | (cronbach* and (alpha or alphas)).tw. | 34399 |
| 74 | (item and (correlation* or selection* or reduction*)).tw. | 37767 |
| 75 | agreement.mp. | 375421 |
| 76 | precision.mp. | 218940 |
| 77 | imprecision.mp. | 10237 |
| 78 | "precise values".mp. | 284 |
| 79 | test-retest.tw. | 36920 |
| 80 | (test and retest).tw. | 39029 |
| 81 | (reliab* and (test or retest)).tw. | 144804 |
| 82 | stability.tw. | 584325 |
| 83 | (interrater or inter-rater or intrarater or intra-rater).tw. | 32063 |
| 84 | (intertester or inter-tester or intratester or intra-tester).tw. | 816 |
| 85 | (interobserver or inter-observer or intraobserver or intra-observer).tw. | 44679 |
| 86 | (intertechnician or inter-technician or intratechnician or intra-technician).tw. | 42 |
| 87 | (interexaminer or inter-examiner or intraexaminer or intra-examiner).tw. | 2372 |
| 88 | (interassay or inter-assay or intraassay or intra-assay).tw. | 13235 |
| 89 | (interindividual or inter-individual or intraindividual or intra-individual).tw. | 51156 |
| 90 | (interparticipant or inter-participant or intraparticipant or intra-participant).tw. | 228 |
| 91 | kappa.tw. | 125695 |
| 92 | kappas.tw. | 1144 |
| 93 | repeatab*.mp. | 55461 |
| 94 | ((replicab* or repeated) and (measure or measures or findings or result or results or test or tests)).mp. | 350022 |
| 95 | (generaliza* or generalisa*).tw. | 66120 |
| 96 | concordance.tw. | 84514 |
| 97 | (intraclass and correlation*).tw. | 37532 |
| 98 | discriminative.tw. | 29026 |
| 99 | "known group".tw. | 1639 |
| 100 | ("factor analysis" or "factor analyses" or "factor structure" or "factor structures").tw. | 73020 |
| 101 | dimension*.tw. | 711659 |
| 102 | subscale*.tw. | 73139 |
| 103 | (multitrait and scaling and (analysis or analyses)).tw. | 157 |
| 104 | "item discriminant*".tw. | 135 |
| 105 | "interscale correlation*".tw. | 180 |
| 106 | (error or errors).tw. | 470247 |
| 107 | "individual variability".tw. | 14147 |
| 108 | "interval variability".tw. | 876 |
| 109 | "rate variability".tw. | 30382 |
| 110 | (variability and (analysis or values)).tw. | 161897 |
| 111 | (uncertainty and (measurement or measuring)).tw. | 10723 |
| 112 | "standard error of measurement".tw. | 2848 |
| 113 | sensitiv*.tw. | 2023065 |
| 114 | responsive*.tw. | 319109 |
| 115 | (limit and detection).tw. | 126828 |
| 116 | "minimal detectable concentration*".tw. | 126 |
| 117 | interpretab*.tw. | 16772 |
| 118 | ((minimal or minimally or clinical or clinically) and (important or significant or detectable) and (change or difference)).tw. | 490324 |
| 119 | (small* and (real or detectable) and (change or difference)).tw. | 14391 |
| 120 | "meaningful change".tw. | 2316 |
| 121 | "ceiling effect".tw. | 2989 |
| 122 | "floor effect".tw. | 1029 |
| 123 | "item response model".tw. | 150 |
| 124 | IRT.tw. | 5131 |
| 125 | rasch.tw. | 6037 |
| 126 | "differential item functioning".tw. | 2417 |
| 127 | DIF.tw. | 5688 |
| 128 | "computer adaptive testing".tw. | 344 |
| 129 | "item bank".tw. | 1003 |
| 130 | "cross-cultural equivalence".tw. | 153 |
| 131 | or/58-130 | 11202904 |
| 132 | (afghanistan or albania or algeria or american samoa or angola or "antigua and barbuda" or antigua or barbuda or argentina or armenia or armenian or aruba or azerbaijan or bahrain or bangladesh or barbados or republic of belarus or belarus or byelarus or belorussia or byelorussian or belize or british honduras or benin or dahomey or bhutan or bolivia or "bosnia and herzegovina" or bosnia or herzegovina or botswana or bechuanaland or brazil or brasil or bulgaria or burkina faso or burkina fasso or upper volta or burundi or urundi or cabo verde or cape verde or cambodia or kampuchea or khmer republic or cameroon or cameron or cameroun or central african republic or ubangi shari or chad or chile or china or colombia or comoros or comoro islands or iles comores or mayotte or democratic republic of the congo or democratic republic congo or congo or zaire or costa rica or "cote d’ivoire" or "cote d’ ivoire" or cote divoire or cote d ivoire or ivory coast or croatia or cuba or cyprus or czech republic or czechoslovakia or djibouti or french somaliland or dominica or dominican republic or ecuador or egypt or united arab republic or el salvador or equatorial guinea or spanish guinea or eritrea or estonia or eswatini or swaziland or ethiopia or fiji or gabon or gabonese republic or gambia or "georgia (republic)" or georgian or ghana or gold coast or gibraltar or greece or grenada or guam or guatemala or guinea or guinea bissau or guyana or british guiana or haiti or hispaniola or honduras or hungary or india or indonesia or timor or iran or iraq or isle of man or jamaica or jordan or kazakhstan or kazakh or kenya or "democratic people’s republic of korea" or republic of korea or north korea or south korea or korea or kosovo or kyrgyzstan or kirghizia or kirgizstan or kyrgyz republic or kirghiz or laos or lao pdr or "lao people's democratic republic" or latvia or lebanon or lebanese republic or lesotho or basutoland or liberia or libya or libyan arab jamahiriya or lithuania or macau or macao or republic of north macedonia or macedonia or madagascar or malagasy republic or malawi or nyasaland or malaysia or malay federation or malaya federation or maldives or indian ocean islands or indian ocean or mali or malta or micronesia or federated states of micronesia or kiribati or marshall islands or nauru or northern mariana islands or palau or tuvalu or mauritania or mauritius or mexico or moldova or moldovian or mongolia or montenegro or morocco or ifni or mozambique or portuguese east africa or myanmar or burma or namibia or nepal or netherlands antilles or nicaragua or niger or nigeria or oman or muscat or pakistan or panama or papua new guinea or new guinea or paraguay or peru or philippines or philipines or phillipines or phillippines or poland or "polish people's republic" or portugal or portuguese republic or puerto rico or romania or russia or russian federation or ussr or soviet union or union of soviet socialist republics or rwanda or ruanda or samoa or pacific islands or polynesia or samoan islands or navigator island or navigator islands or "sao tome and principe" or saudi arabia or senegal or serbia or seychelles or sierra leone or slovakia or slovak republic or slovenia or melanesia or solomon island or solomon islands or norfolk island or norfolk islands or somalia or south africa or south sudan or sri lanka or ceylon or "saint kitts and nevis" or "st. kitts and nevis" or saint lucia or "st. lucia" or "saint vincent and the grenadines" or saint vincent or "st. vincent" or grenadines or sudan or suriname or surinam or dutch guiana or netherlands guiana or syria or syrian arab republic or tajikistan or tadjikistan or tadzhikistan or tadzhik or tanzania or tanganyika or thailand or siam or timor leste or east timor or togo or togolese republic or tonga or "trinidad and tobago" or trinidad or tobago or tunisia or turkey or turkmenistan or turkmen or uganda or ukraine or uruguay or uzbekistan or uzbek or vanuatu or new hebrides or venezuela or vietnam or viet nam or middle east or west bank or gaza or palestine or yemen or yugoslavia or zambia or zimbabwe or northern rhodesia or global south or africa south of the sahara or sub-saharan africa or subsaharan africa or africa, central or central africa or africa, northern or north africa or northern africa or magreb or maghrib or sahara or africa, southern or southern africa or africa, eastern or east africa or eastern africa or africa, western or west africa or western africa or west indies or indian ocean islands or caribbean or central america or latin america or "south and central america" or south america or asia, central or central asia or asia, northern or north asia or northern asia or asia, southeastern or southeastern asia or south eastern asia or southeast asia or south east asia or asia, western or western asia or europe, eastern or east europe or eastern europe or developing country or developing countries or developing nation? or developing population? or developing world or less developed countr* or less developed nation? or less developed population? or less developed world or lesser developed countr* or lesser developed nation? or lesser developed population? or lesser developed world or under developed countr* or under developed nation? or under developed population? or under developed world or underdeveloped countr* or underdeveloped nation? or underdeveloped population? or underdeveloped world or middle income countr* or middle income nation? or middle income population? or low income countr* or low income nation? or low income population? or lower income countr* or lower income nation? or lower income population? or underserved countr* or underserved nation? or underserved population? or underserved world or under served countr* or under served nation? or under served population? or under served world or deprived countr* or deprived nation? or deprived population? or deprived world or poor countr* or poor nation? or poor population? or poor world or poorer countr* or poorer nation? or poorer population? or poorer world or developing econom* or less developed econom* or lesser developed econom* or under developed econom* or underdeveloped econom* or middle income econom* or low income econom* or lower income econom* or low gdp or low gnp or low gross domestic or low gross national or lower gdp or lower gnp or lower gross domestic or lower gross national or lmic or lmics or third world or lami countr* or transitional countr* or emerging economies or emerging nation?).ti,ab,sh,kf. | 2693244 |
| 133 | (afghan or afghans or afghani or albanian? algerian? or american samoan? or angolan? or antiguan? or barbudan? or argentine? or argentinian? or argentinean? or armenian? or aruban? or azerbaijani? or bahraini? or bangladeshi? or bangalees or bajan? or belarusian? or byelorussian? or belizean? or beninese? or bhutanese or bolivian? or bosnian? or botswana or batswana or brazilian? or brasilian? or bulgarian? or burkinabe or burkinese or burundian? or cape verdean? or cabo verdean? or cambodian? or khmer or cameroonian? or central african? or chadian? or chilean? or chinese or colombian? or comorian? or congolese or costa rican? or ivorian? or croatian? or cuban? or cypriot? or czech? or djiboutian? or dominican? or ecuadorian? or egyptian? or salvadoran? or equatorial guinean? or equatoguinean? or eritrean? or estonian? or swazi? or swati? or ethiopian? or fijian or gabonese or gabonaise or gambian? or georgian? or ghanaian? or gibraltarian? or greek? or grenadian? or guamanian? or guatemalan? or guinean? or bissau guinean? or guyanese or haitian? or honduran? or hungarian? or indian? or indonesian? or iranian? or iraqian? or iraqi? or manx or jamaican? or jordanian? or kazakhstani? or kenyan? or kirabati or kirabatian? or north korean? or korean? or kosovar? or kosovan? or kyrgyz* or lao or laotian? or latvian? or lebanese or lesothan? or lesothonian? or mosotho or basotho or liberian? or libyan? or lithuanian? or macanese or macedonian? or malagasy or madagascan? or malawian? or malaysian? or maldivian? or malian? or maltese or marshallese? or mauritanian? or mauritian? or mexican? or micronesian? or moldovan? or mongolian? or mongol or montenegrin? or moroccan? or mozambican? or burmese or myanma or namibian? or nauruan? or nepali or nepalese or netherlands antillean? or nicaraguan? or nigerien? or nigerian? or northern mariana islander? or mariana? or omani? or pakistani? or palauan? or panamanian? or papua new guinean? or paraguayan? or peruvian? or philippine? or philipine? or phillipine? or phillippine? or filipino? or filipina? or polish or pole or poles or portuguese or puerto rican? or romanian? or russian? or soviet people or soviet population or rwandan? or rwandese or ruandan? or ruandese or samoan? or sao tomean? or santomean? or saudi arabian? or saudi? or senegalese or serbian? or montenegrin? or seychellois or seychelloise? or sierra leonean? or slovak? or slovene? or solomon islander? or somali? or south african? or south sudanese or sri lankan? or ceylonese or kittitian? or nevisian? or saint lucian? or vincentian? or sudanese or surinamese? or syrian? or tajik? or tajikistani? or tanzanian? or tanganyikan? or thai or timorese? or togolese or tongan? or trinidadian? or tobagonian? or tunisian? or turk? or turkish or turkmen? or tuvaluan? or ugandan? or ukrainian? or uruguayan? or uzbek? or vanuatu* or venezuelan? or vietnamese or yemeni? or yemenite? or yemenese or yugoslav? or yugoslavian? or zambian? or zimbabwean?).ti,ab,sh,kf. | 1389019 |
| 134 | 132 or 133 | 3428452 |
| 135 | 3 and 27 and 57 | 42587 |
| 136 | 3 and 27 and 57 and 131 | 18063 |
| 137 | 3 and 27 and 57 and 131 and 134 | 4772 |
| 138 | limit 137 to english language | 4527 |

| **CINAHL Search Strategy** | |  | |
| --- | --- | --- | --- |
| **#** | **Query** | | **Results** |
| S1 | (MH "Infant, Newborn") OR (MH "Infant") | | 271,495 |
| S2 | "infant" OR "newborn" OR "newborn infant" OR "neonatal" OR "neonat*" OR "newborn" | | 343,384 |
| S3 | S1 OR S2 | | 343,384 |
| S4 | (MH "Community Health Workers") | | 4,158 |
| S5 | "community health worker*" OR "primary health worker*" OR "health volunteer*" OR "health worker*" OR "front#line" | | 20,111 |
| S6 | (MH "Nursing Assessment") | | 18,452 |
| S7 | (MH "Neonatal Assessment") | | 4,281 |
| S8 | "newborn screening" OR "neonatal screening" | | 2,669 |
| S9 | "Integrated Management of Childhood Illness*" OR "IMCI" OR ( "Integrated Management of Newborn and Childhood Illness*" ) OR "IMNCI" OR "danger sign*" OR "Young Infant* Study" OR "Young Infant* Sign*" OR "Young Infant* Clinical Sign*" OR "Young Infant* Clinical Sign* Study" | | 993 |
| S10 | (MH "Algorithms") OR ""algorithm*"." | | 68,743 |
| S11 | (MH "Child Health Services") | | 8,486 |
| S12 | (MH "Risk Assessment") OR ""risk assessment"" | | 141,289 |
| S13 | ""clinical decision"" | | 17,389 |
| S14 | S4 OR S5 OR S6 OR S7 OR S8 OR S9 OR S10 OR S11 OR S12 OR S13 | | 272,629 |
| S15 | "illness" OR "disease" OR "survival" OR "infection" | | 1,448,954 |
| S16 | (MH "Infection") | | 15,402 |
| S17 | ""bacterial infections and mycoses"" | | 588 |
| S18 | (MH "Sepsis") OR (MH "Neonatal Sepsis") | | 19,709 |
| S19 | "septicemia" OR "sepsis" | | 34,408 |
| S20 | ("possible serious bacterial infection" or "possible severe bacterial infection" or "PSBI" or "serious bacterial infection" or "SBI" | | 720 |
| S21 | "serious illness*" or "severe illness*" or "severe disease*" or "serious disease*" | | 9,394 |
| S22 | "sick infant*" OR "sick young infant*" OR "sick newborn*" OR "sick neonate*" OR "mortality" | | 341,283 |
| S23 | (MH "Communicable Diseases") | | 12,410 |
| S24 | "communicable disease*" OR "communicable" nn2 "disease*" | | 17,484 |
| S25 | (MH "Bacteremia") OR "bacter?emia" | | 7,034 |
| S26 | (MH "Meningitis") OR "meningitis" | | 10,795 |
| S27 | (MH "Urinary Tract Infections") OR ""urinary tract infection"" | | 16,186 |
| S28 | (MH "Pneumonia") OR "pneumonia" | | 48,535 |
| S29 | "acute" and "lower" and "respiratory infection" | | 287 |
| S30 | (MH "Respiratory Tract Infections") OR ""respiratory tract infection"" | | 12,163 |
| S31 | (MH "Patient Admission") OR (MH "Hospitalization") | | 62,406 |
| S32 | "hospitali#*" or "admit*" or "admission*" | | 253,647 |
| S33 | S15 OR S16 OR S17 OR S18 OR S19 OR S20 OR S21 OR S22 OR S23 OR S24 OR S25 OR S26 OR S27 OR S28 OR S29 OR S30 OR S31 OR S32 | | 1,786,863 |
| S34 | (MH “Psychometrics”) or ( TI psychometr* or AB psychometr* ) or ( TI clinimetr* or AB clinimetr* ) or ( TI clinometr* OR AB clinometr* ) or (MH “Outcome Assessment”) or ( TI outcome assessment or AB outcome assessment ) or ( TI outcome measure* or AB outcome measure* ) or (MH “Health Status Indicators”) or (MH “Reproducibility of Results”) or (MH “Discriminant Analysis”) or ( ( TI reproducib* or AB reproducib* ) or ( TI reliab* or AB reliab* ) or ( TI unreliab* or AB unreliab* ) ) or ( ( TI valid* or AB valid* ) or ( TI coefficient or AB coefficient ) or ( TI homogeneity or AB homogeneity ) ) or ( TI homogeneous or AB homogeneous ) or ( TI “coefficient of variation” or AB “coefficient of variation” ) or ( TI “internal consistency” or AB “internal consistency” ) or (MH “Internal Consistency+”) or (MH “Reliability+”) or (MH “Measurement Error+”) or (MH “Content Validity+”) or “hypothesis testing” or “structural validity” or “cross-cultural validity” or (MH “Criterion-Related Validity+”) or “responsiveness” or “interpretability” or ( TI reliab* or AB reliab* ) and ( (TI test or AB test) OR (TI retest or AB retest) ) or ( TI stability or AB stability ) or ( TI interrater or AB interrater ) or ( TI inter-rater or AB inter-rater ) or ( TI intrarater or AB intrarater ) or ( TI intra-rater or AB intrarater ) or ( TI intertester or AB intertester) or (TI inter-tester or AB inter-tester) or ( TI intratester or AB intratester) or ( TI intra-tester or AB intra-tester) or ( TI interobserver or AB interobserver) or (TI inter-observer or AB inter-observer ) or ( TI intraobserver or AB intraobserver) or ( TI intra-observer or AB intra-observer) or ( TI intertechnician or AB intertechnician) or (TI inter-technician or AB inter-technician) or ( TI intratechnician or AB intratechnician ) or ( TI intra-technician or AB intra-technician ) or ( TI interexaminer or AB interexaminer ) or (TI inter-examiner or AB inter-examiner) or (TI intraexaminer or AB intraexaminer ) OR (TI intra-examiner or AB intra-examiner ) or (TI intra-examiner or AB intraexaminer ) or (TI interassay or AB interassay ) or ( TI inter-assay or AB inter-assay ) or ( TI intraassay or AB intraassay) or ( TI intra-assay or AB intra-assay ) or (TI interindividual or AB interindividual) or (TI inter-individual or AB inter-individual) OR (TI intraindividual or AB intraindividual) or (TI intra-individual or AB intra-individual) or (TI interparticipant or AB interparticipant) or (TI inter-participant or AB inter-participant ) or (TI intraparticipant or AB intraparticipant) or (TI intra-participant or AB intra-participant ) or (TI kappa or AB kappa) or (TI kappa’s or AB kappa’s ) or (TI kappas or AB kappas) or (TI repeatab* or AB repeatab*) or ( TI responsive* or AB responsive* ) or ( TI interpretab* or AB interpretab* ) | | 687,847 |
| S35 | (MH "Developing Countries") OR ( (Africa or Asia or Caribbean or West Indies or South America or Latin America or Central America) ) OR ( (Afghanistan or Albania or Algeria or Angola or Antigua or Barbuda or Argentina or Armenia or Armenian or Aruba or Azerbaijan or Bahrain or Bangladesh or Barbados or Benin or Byelarus or Byelorussian or Belarus or Belorussian or Belorussia or Belize or Bhutan or Bolivia or Bosnia or Herzegovina or Hercegovina or Botswana or Brasil or Brazil or Bulgaria or Burkina Faso or Burkina Fasso or Upper Volta or Burundi or Urundi or Cambodia or Khmer Republic or Kampuchea or Cameroon or Cameroons or Cameron or Camerons or Cape Verde or Central African Republic or Chad or Chile or China or Colombia or Comoros or Comoro Islands or Comores or Mayotte or Congo or Zaire or Costa Rica or Cote d'Ivoire or Ivory Coast or Croatia or Cuba or Cyprus or Czechoslovakia or Czech Republic or Slovakia or Slovak Republic or Djibouti or French Somaliland or Dominica or Dominican Republic or East Timor or East Timur or Timor Leste or Ecuador or Egypt or United Arab Republic or El Salvador or Eritrea or Estonia or Ethiopia or Fiji or Gabon or Gabonese Republic or Gambia or Gaza or Georgia Republic or Georgian Republic or Ghana or Gold Coast or Greece or Grenada or Guatemala or Guinea or Guam or Guiana or Guyana or Haiti or Honduras or Hungary or India or Maldives or Indonesia or Iran or Iraq or Isle of Man or Jamaica or Jordan or Kazakhstan or Kazakh or Kenya or Kiribati or Korea or Kosovo or Kyrgyzstan or Kirghizia or Kyrgyz Republic or Kirghiz or Kirgizstan or Lao PDR or Laos or Latvia or Lebanon or Lesotho or Basutoland or Liberia or Libya or Lithuania or Macedonia or Madagascar or Malagasy Republic or Malaysia or Malaya or Malay or Sabah or Sarawak or Malawi or Nyasaland or Mali or Malta or Marshall Islands or Mauritania or Mauritius or Agalega Islands or Mexico or Micronesia or Middle East or Moldova or Moldovia or Moldovian or Mongolia or Montenegro or Morocco or Ifni or Mozambique or Myanmar or Myanma or Burma or Namibia or Nepal or Netherlands Antilles or New Caledonia or Nicaragua or Niger or Nigeria or Northern Mariana Islands or Oman or Muscat or Pakistan or Palau or Palestine or Panama or Paraguay or Peru or Philippines or Philipines or Phillipines or Phillippines or Poland or Portugal or Puerto Rico or Romania or Rumania or Roumania or Russia or Russian or Rwanda or Ruanda or Saint Kitts or St Kitts or Nevis or Saint Lucia or St Lucia or Saint Vincent or St Vincent or Grenadines or Samoa or Samoan Islands or Navigator Island or Navigator Islands or Sao Tome or Saudi Arabia or Senegal or Serbia or Montenegro or Seychelles or Sierra Leone or Slovenia or Sri Lanka or Ceylon or Solomon Islands or Somalia or South Africa or Sudan or Suriname or Surinam or Swaziland or Syria or Tajikistan or Tadzhikistan or Tadjikistan or Tadzhik or Tanzania or Thailand or Togo or Togolese Republic or Tonga or Trinidad or Tobago or Tunisia or Turkey or Turkmenistan or Turkmen or Uganda or Ukraine or Uruguay or USSR or Soviet Union or Union of Soviet Socialist Republics or Uzbekistan or Uzbek or Vanuatu or New Hebrides or Venezuela or Vietnam or Viet Nam or West Bank or Yemen or Yugoslavia or Zambia or Zimbabwe or Rhodesia) ) OR ( ((developing or less* developed or under developed or underdeveloped or middle income or low* income or underserved or under served or deprived or poor*) N1 (countr* or nation? or population? or world)) ) OR ( ((developing or less* developed or under developed or underdeveloped or middle income or low* income) N1 (economy or economies)) ) OR ( (low* N1 (gdp or gnp or gross domestic or gross national)) ) OR (low N3 middle N3 countr*) OR ( (lmic or lmics or third world or lami countr*) ) OR transitional countr* OR ( ((high burden or high-burden or countdown) N1 countr*) ) | | 649,367 |
| S36 | S3 AND S14 AND S33 | | 9,899 |
| S37 | S3 AND S14 AND S33 AND S34 | | 1,536 |
| S38 | S3 AND S14 AND S33 AND S34 AND S35 | | 344 |
